# Implementing Machine Learning Models for Prediction of Gender-Affirming Mastectomy Complications: Estimating Performance and Accuracy

**DOI:** 10.1093/asjof/ojaf143

**Published:** 2025-11-01

**Authors:** Ron Skorochod, Stav Ben-Tov, Roy Hanan Wolf, Yoram Wolf

## Abstract

**Background:**

Machine learning (ML) gained recent popularity because of its usefulness and applicability in medicine. Surgical specialties utilize ML for patient selection, optimization, and prediction of outcomes.

**Objectives:**

The aim of the authors of this study is to develop, validate, and compare ML algorithms for prediction of gender-affirming mastectomy complications.

**Methods:**

Analysis of gender-affirming mastectomies performed by the senior author was performed retrospectively. Six ML algorithms were trained and optimized based on a portion of the data and tested on the remainder. Models were compared in accuracy of prediction, sensitivity, specificity, positive predictive value (PPV), and negative predictive value (NPV). Receiver operating characteristic (ROC) curves were computed for each model, and the area under the curve (AUC) was calculated.

**Results:**

A total of 268 patients comprised the entire dataset, of which 214 were utilized to train the models. Random forest (RF) and K-nearest neighbors demonstrated the highest model accuracies of 92.6%, closely followed by XGBoost with 90.7%, and neural networks and support vector machine with 88.9%. Logistic regression recorded the lowest final accuracy of 87.0%. Sensitivity ranged from 61.90% for the K-nearest neighbors model to 90.48% for the neural networks model, whereas specificity reached 100% in RF and XGBoost. Logistic regression, RF, and support vector machine showed strong PPV and NPV metrics. AUC score was led by RF, at 0.904.

**Conclusions:**

RF demonstrated the highest accuracy and AUC, with similarly high specificity value and PPV. Application of ML algorithms may be useful for predicting gender-affirming mastectomy complications and could aid surgeons in patient selection.

**Level of Evidence: 5 (Therapeutic):**

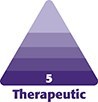

Gender-affirming mastectomy is a key step in the gender confirmation process of female to male individuals. Associated with a marked improvement in quality of life, the procedure represents the pivotal step in the transition process and is highly sought after.^1,2^

Despite its benefits, the procedure carries a measurable risk of postoperative complications. Reported rates vary across studies, with hematoma and seroma being the most commonly reported complications, occurring in ∼5% to 25% of patients.^1-4^ These complications can delay recovery, necessitate reoperation, and adversely affect patient satisfaction. Understanding and predicting which patients are at greatest risk remains a key challenge in perioperative planning.

As with any elective surgery, gender-affirming mastectomy requires careful evaluation of patient candidacy and assessment of their risk for postoperative complications.

Current literature contains ample reports of safety and potential risk factors related to baseline and surgical characteristics of candidates.

Age, BMI, race, medical history, and hormonal therapy are only the tip of the iceberg when assessing variables that can impact surgical outcomes and must be assessed for each individual patient.^5-8^

Current literature commonly focuses on hematomas, seromas, and skin and nipple ischemia when discussing complications of gender-affirming mastectomies, mainly because of its relative frequency and harmful consequences.

Previous reports highlighted the association of age, obesity, race, and surgical technique as potential risk factors for the development of these complications.^8-11^

Current predictive models and research data lack a holistic and comprehensive approach when assessing risk for complications in gender-affirming mastectomies. Studies tend to investigate the impact of individual factors on the risk for complications, rather than create multifaceted models that assess and determine surgical safety and risk for complications.

Machine learning (ML) offers an opportunity to improve complication risk prediction by analyzing large datasets and identifying complex relationships between multiple variables. Unlike traditional statistical models, which require predefined assumptions about variable relationships, ML algorithms can automatically learn patterns from data, adapt to new information, and improve prediction accuracy over time. In surgery, ML has been successfully applied to predict outcomes such as readmission after colorectal surgery, mortality following cardiac procedures, and surgical site infections in orthopedic operations.^12-14^

Gender-affirming surgery, especially chest masculinization procedures, has yet to be investigated in relation to ML applicability.

Incorporation of artificial intelligence (AI) models in the evaluation of patients at risk for surgical complications in gender-affirming procedures is of great interest; with the heterogenic population seeking these procedures, it can be difficult to identify patients at risk. Timely recognition of these patients can aid surgeons in tailoring the surgical procedures to the patient and thus increase their safety and the patient's satisfaction.

The primary aim of the authors of this study is to evaluate and compare the performance of various ML algorithms in predicting postoperative complications following gender-affirming mastectomy. The secondary aim was to identify which patient and surgical variables contributed most to model predictions, thereby providing insight into key risk factors in this population. By designing the models and proofing their concept, we anticipate physicians to understand the importance and potential of AI integration in transgender healthcare and improve patient safety and satisfaction through more tailored surgical planning.

## METHODS

### Ethical Considerations

The study has been approved by the local institution’s (Hillel Yaffe Medical Center) review board, and all researchers adhered meticulously to the approved protocol.

### Study Population

All patients who underwent gender-affirming mastectomies by the senior author (Y.W.) during the years 2003-2025 were reviewed for inclusion in the study.

Participants were deemed eligible for inclusion in the study if they underwent isolated mastectomies, without concomitant procedures, and fulfilled the mandatory postoperative follow-up period of 30 days. Patients were excluded from the study if they did not fulfill the required follow-up period or underwent simultaneous surgical procedures.

### Surgical Technique

This trial analyzes results of 4 surgical approaches: the periareolar approach, the omega-shaped resection (also termed nipple-areolar complex (NAC) on scar), the spindle-shaped mastectomy with NAC flap, and the free NAC graft mastectomy.

The periareolar approach involves cutting around and reducing the areola and creating a dermal flap to support the nipple–areola complex. The breast pocket is dissected through this incision, and the skin is reduced around the areola.

The omega-shaped resection extends the incision medially and laterally, and finally a horizontal suture line.

The spindle-shaped mastectomy leaves a skin flap inferiorly, on which the nipple–areola complex is based. A round skin excision is made superiorly at the site of the new nipple. The nipple–areola complex is inserted through the opening and sutured with a horizontal suture line.

Free NAC involves a complete spindle-shaped mastectomy with a free nipple–areola graft.

The selection of mastectomy type was determined by the surgeon based on patient-specific factors, including chest size and contour, skin elasticity, degree of ptosis, and patient preference. Although the decision was ultimately made by the surgeon, it was conducted in discussion with the patient to ensure alignment of the surgical technique with both anatomical considerations and aesthetic goals.

### Data Preparation

Medical files and surgical notes were evaluated for relevant variables. Demographic, medical comorbidities, intraoperative details, and postoperative outcomes were extracted into an electronic data sheet. Imputation based on mean scores was utilized to account for missing data. Instances where missing data accounted for >10% of total data lead to removal from future analysis. The variables extracted from the medical records and utilized for model development included age, BMI, hypertension status, diabetes mellitus, asthma, previous testosterone therapy, history of breast surgery, surgical technique (periareolar, NAC on scar, NAC flap, or free NAC), and average resection weight. These variables are summarized in Table 1. Predictive variables were selected a priori based on clinical relevance, availability in medical records, and consistency across patients.

**Table 1.** Baseline Characteristics of Cohort Patients, Stratified Based on Development of Postoperative Complications

Variable	Complications (*n* = 106)	No complications (*n* = 162)	*P*-value
Age (years)	22.08 + 6.8	20.66 + 6.6	.13
BMI (kg/m^2^)	25.26 + 5.28	26.64 + 4.24	.21
Hypertension	0 (0%)	1 (0.6%)	1.00
Diabetes mellitus	1 (0.9%)	2 (1.2%)	.50
Asthma	7 (6.6%)	6 (3.6%)	.31
Psychiatric	5 (4.7%)	7 (4.3%)	.90
Fibromyalgia	1 (0.9%)	3 (1.8%)	.90
Epilepsy	0 (0%)	0 (0%)	1.01
Hypothyroidism	4 (3.8%)	6 (3.6%)	.90
Thrombophilia	0 (0%)	0 (0%)	1.00
Inflammatory bowel disease	7 (6.6%)	11 (6.8%)	.91
Migraine	20 (18.9%)	24 (14.9%)	.80
Pre-op Hb (g/dL), mean + SD	12.1 + 1.4	12.3 + 1.7	.31
Pre-op WBC (10^3^/µL), mean + SD	7.6 + 2.4	7.7 + 1.1	.72
Pre-op platelet count (10^3^/µL), mean + SD	312.4 + 117	288. 8 + 130	.11
Pre-op glucose (mg/dL), mean + SD	87 + 19	90 + 11	.11
Previous testosterone therapy	58 (54.7%)	60 (37%)	.03*
Previous breast surgery	2 (1.9%)	2 (1.2%)	.21
Surgical technique			.0001*
Periareolar	28 (26.4%)	0	
NAC on scar	5 (4.7%)	0	
NAC flap	53 (50%)	0	
Free NAC	20 (19%)	162 (100%)	
Average resection weight (g), mean + SD	348 + 257	574 + 358	.0001*
Average liposuction volume (mL), mean + SD	250 + 110	325 + 100	.0001*

Statistical significance is indicated by an asterisk (*) next to the *P*-value. SD, standard deviation; WBC, white blood cell.

### Outcome Definition

The primary outcome examined in this study was a composite outcome of complications. Complications included in the analysis were limited to those observed in the study cohort within 30 days postoperatively and included hematoma, seroma, surgical site infection, flap necrosis, and wound dehiscence. Outcome was binarily defined and was queried as such in the model training and testing phases. In our study, the unit of analysis refers to the patient and not to each breast individually.

### Data Segmentation

Data segmentation was performed as follows: the complete dataset was randomly divided into a training dataset (comprising 80% of data) and a testing dataset (comprising 20% of data). Division of the data allowed for the development and validation of the models in a controlled, reproducible environment. The training and testing datasets presented to each ML model were the same.

### Model Development

Six ML models were developed using the following algorithms: logistic regression, K-nearest neighbors (KNNs), neural networks, random forest (RF), XGBoost (XGB), and support vector machine (SVM). Models were chosen for their efficacy in handling binary classification tasks and their common use in medical literature for similar outcome analyses.

### Five-Fold Cross Validation

Five-fold cross validation is a technique that involves dividing the “training” dataset into 5 equal parts. Every validation cycle, 4 of the parts are utilized to train the model, whereas the fifth part is reserved to serve as the “test” dataset. The process is repeated 5 times, ensuring each part of the dataset serves as a testing dataset once. Accuracy is measured for each cycle, and the mean score is regarded as the overall accuracy of the cross-validation process.

In this presented research, we applied the 5-fold cross-validation process at the training stage for each individual ML algorithm.

### Hyperparameter Tuning

Hyperparameters were tuned to maximal performance accuracy, as per the 5-fold cross-validation outcomes, for each of the models. Accurate and complete hyperparameters utilized for final model performance evaluation can be seen in the Appendix.

### Model Testing

Following model training and hyperparameter tuning, the optimized models were applied to the reserved testing dataset, which represents 20% of the overall dataset. Receiver operating characteristic (ROC) curves were computed, and the area under the curve (AUC) was calculated alongside the 95% CI.

**Figure 1.**
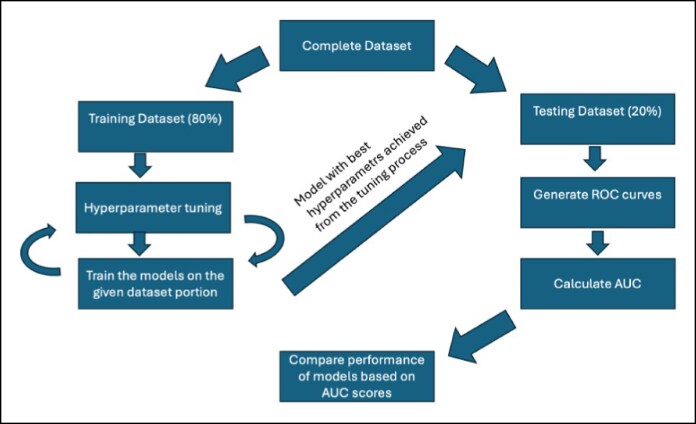
Flow chart demonstrating the training and testing process of the machine learning models.

Model accuracy was evaluated and was utilized to compare the models' effectiveness in addition to the AUC. Figure 1 demonstrates the training and testing process of the ML models in a flowchart manner.

### Feature Importance

A feature importance analysis was performed to further explore and interpret the chosen model by ranking and visualizing the relative impact of each variable on outcome prediction.

All ML model development, including training, hyperparameter tuning, and performance evaluation, was performed between March 2025 and June 2025 on the finalized dataset covering surgeries from 2003 to 2025.

### Statistical Analysis

All analyses were performed using Python with scikit, matplotlib, and pandas libraries.

## RESULTS

A total of 268 patients underwent gender-affirming mastectomy and were included in the study. Of these, 214 patients (80%) comprised the training dataset, and 54 patients (20%) comprised the testing dataset for final model performance evaluation.

Two-hundred and sixty-eight patients were included in the complete dataset, of which 106 (39.5%) experienced at least 1 complication of the composite outcome. The breakdown of complications was as follows: 23 cases of seroma (22%), 32 cases of hematoma (30%), 5 cases of surgical site infection (5%), 1 case of flap necrosis (1%), and 45 cases of wound dehiscence (42%).

At baseline, patients were found to be similar at most variables, with statistically significant differences being noted only in stratification of surgical techniques, weight of flap resection, volume of liposuction, and frequency of previous testosterone therapy. Complete analysis and comparison of variables can be seen under Table 1.

It should be noted that in our dataset, all patients without complications were managed with the free NAC technique, whereas the other surgical techniques (periareolar, NAC-on-scar, NAC flap) were only observed in the complication cohort.

### Model Performance and Accuracy

Neural networks achieved a cross-validation accuracy of 80.87% with a final model accuracy of 88.9%. SVM achieved a cross-validation accuracy of 86.02% and a final accuracy of 88.9%. RF achieved a cross-validation accuracy of 88.82% and a final accuracy of 92.6. XGB achieved a cross-validation accuracy of 90.21%, with final model accuracy of 90.70%. KNNs achieved a 79.50% cross-validation accuracy and a 92.6% final accuracy. Logistic regression achieved a cross-validation accuracy of 83.20% and a final model accuracy of 87.0%.

Final model accuracy is summarized in Table 2.

**Table 2.** Models Accuracy, Area Under Curve, Sensitivity, Specificity, Positive Predictive Value, and Negative Predictive Value

Model	Sensitivity (%)	Specificity (%)	PPV (%)	NPV (%)	Accuracy	AUC (95% CI)
Logistic regression	80.95	90.91	85	88.24	0.87	0.855 (0.743-0.955)
Random forest	80.95	100	100	89.19	0.926	0.904 (0.823-0.976)
XGBoost	76.19	100	100	86.84	0.907	0.884 (0.783-0.971)
K-Nearest neighbors	61.90	96.97	92.85	80	0.833	0.791 (0.675-0.886)
Neural networks	90.48	87.88	82.61	93.55	0.889	0.890 (0.795-0.967)
Support vector machine	85.71	90.91	85.71	90.91	0.889	0.881 (0.781-0.966)

AUC, area under the curve; NPV, negative predictive value; PPV, positive predictive value.

### Confusion Matrixes

Sensitivity, specificity, positive predictive value (PPV), and negative predictive value were calculated using a predicted vs actual outcomes table (also known as a confusion matrix).

Neural networks demonstrated the highest sensitivity (90.48%), whereas RF and XGB achieved 100% specificity and PPV. SVM provided a balanced performance across all metrics.

Complete and detailed results are depicted in Table 2.

### Receiver Operating Characteristic Curve Analysis

ROC curves were computed for each model, and the AUC was calculated with a 95% CI and are summarized alongside all model outcome parameters in Table 2.

Overall, the highest discriminative performance was observed for the RF model with an AUC of 0.904 (95% CI, 0.82-0.98), closely followed by neural networks and XGB.

**Figure 2.**
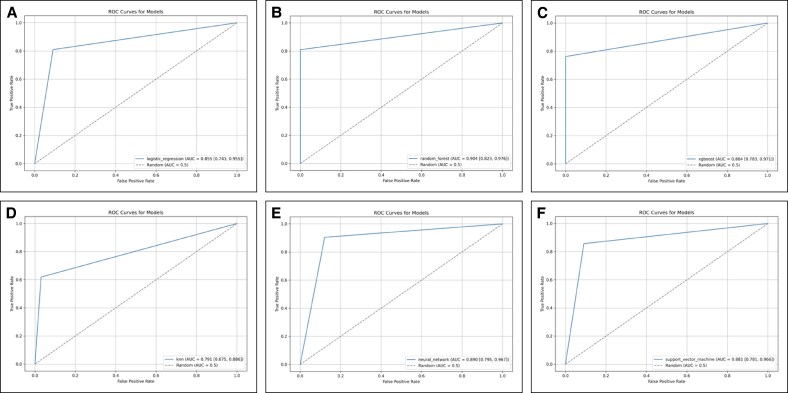
Receiver operating characteristic curve of (A) the logistic regression model, (B) random forest model, (C) XGBoost model, (D) K-nearest neighbors model, (E) neural networks model, and (F) the support vector machine model.

All ROC and corresponding AUC are shown in Figure 2.

### Feature Importance Analysis

Feature importance analysis was followed. Interpretation of its results highlights surgical technique, resection weight, age, and preoperative hemoglobin levels, to be the variables with the greatest impact on outcome development.

**Figure 3.**
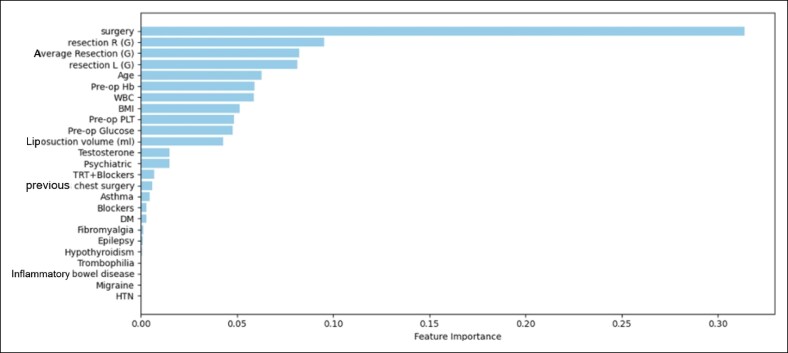
Feature importance analysis of the random forest model.

A complete feature analysis graph depiction is shown in Figure 3.

## DISCUSSION

Integration of ML in surgery provides hypothetical and practical applications, ranging from predicting patients at risk for complications, prolonged hospital stays, and opioid dependency to patient education and characterization.

Simpson et al reported their positive experience with the application of the RF algorithm on a database of patients undergoing orthopedic spine surgery with the aim of predicting opioid dependency.^15^ Tseng et al similarly concluded RF is the optimal model to predict acute kidney injury in patients following cardiac surgery.^16^

Jurgenseimer et al reported their experience with ML prediction of secondary meniscal tears after anterior cruciate ligament reconstruction.^17^ In addition to the author’s conclusion that, once again, RF is the optimal algorithm, integration of ML into daily practice can provide real-time risk estimation for counseling and timely intervention to guide patient expectations.

Breast surgery research has joined the medical community in pursuit of ML integration. Common areas of focus, such as patient-reported outcomes and postoperative adverse events have been reported to integrate ML with satisfactory results. Chen et al trained 5 ML models to predict improvement of BREAST-Q scores.^18^ The trained models were later validated on an external database and compared. Naoum et al, Braun et al, and Chen et al reported success with the prediction of a wide range of complications related to breast reconstruction and advocate for its expansion to the general practice.^19-21^

The potential of ML in gender-affirming care extends beyond the prediction of complications and critically analyzes psychological and sexual outcomes. Kundu et al trained a RF model to predict suicidal thoughts and acceptance of help for mental health and substance abuse in sexual and gender minority young adults with great success, achieving satisfactory results.^22,23^ Moody et al sought to identify the association between brain connectivity and body congruence after cross-sex hormone therapy.^24^

ML applications can aid in real-time risk estimation and recognition of complication-prone patients, thereby enhancing the procedure's safety and appropriately regulating patient expectations.

Ganguli et al experimented with ML for prediction of hypertension in transgender patients undergoing gynecologic surgery.^25^ The authors suggested that drawing conclusions based on models trained on cisgender patients can impair inference of results and lead to inaccurate risk estimation.

In our study, RF achieved the greatest final model accuracy of 92.6 and AUC of 0.904 (95% CI, 0.823-0.976). Specificity and PPV were also greatest for the RF model, with the optimal score of 100%. However, sensitivity appeared lower with 80.95%. Although the model provides extravagant parameters in terms of accuracy, in instances where sensitivity is the greatest concern neural networks provide an adequate option with a sensitivity of 90.48% and comparable AUC of 0.89 (95% CI, 0.795-0.967). Potential instances where this would be important include the prediction of high-risk complications that may result in reoperation or morbidity, where the risk of generating a false positive outweighs that of a false negative classification.

In addition to model performance, our analysis also demonstrated significant baseline differences between the complication and no-complication cohorts (Table 1). Surgical technique, resection weight, liposuction volume, and previous testosterone therapy differed significantly between the groups. These findings highlight that both technical and patient-related characteristics may predispose patients to complications and should be considered alongside model predictions when counseling patients and planning surgery.

Feature importance analysis further emphasized the clinical relevance of surgical technique, resection weight, age, and preoperative hemoglobin levels as key predictors of postoperative complications. These findings suggest that such variables may be incorporated into preoperative risk stratification and patient counseling for individuals undergoing gender-affirming mastectomy. For example, patients with larger resection weights or lower preoperative hemoglobin may warrant closer monitoring, whereas choice of surgical technique may be guided by both complication risk and aesthetic considerations.

Applicability of ML in clinical practice requires careful understanding of the various models and their ability in data classification and prediction. Relying on inappropriate models for general prediction can lead to subpar performance and clinical use.

Logistic regression focuses on outcome odds, which are defined as the ratio between the probability that an event will occur and the probability that it will not occur. It adjusts the output to have a value between 0 and 1, representing the probability that the event will occur given a specific set of input variables. This model is not only helpful for prediction and classification but also helps examine the effect of each input variable on the odds of the outcome by analyzing odds ratio, which are the ratios between odds.

SVM aims to create a decision boundary, called a hyperplane, between classes in a specific feature space. This hyperplane is utilized for classification by determining on which side of the hyperplane the input instance is.

KNNs differ as for the classification of a new set of input variables, it finds the closest K instances from the training set by calculating distances based on the input variables. Then, to decide on a classification, it uses the majority rule between the closest K instances of the training set.

Neural networks' basic unit is the artificial neuron, which receives an input, applies an activation function to it, and outputs the result to the next neuron. The neurons are organized in layers where each neuron is connected to all the neurons of the next layer. Each connection is weighted, reflecting the strength of the connection between the 2 neurons.

RF and XGB are ensemble models, consists of a collection of decision tree classifiers. Each tree is a classification or regression tree, which is designed to classify an input using a tree-structured set of rules; each rule is an if-then rule that splits the data. This is done repeatedly to create branches where the final rule in each branch decides on the classification. Although RF uses bagging (bootstrap aggregating) to train multiple parallel classifiers. creating different random subsets of the training data, fitting a decision tree to each subset, and then combining their predictions to reduce variance and improve stability, XGB uses gradient boosting, where trees are built sequentially, with each tree trying to correct the errors made by the previous one.

Because our dataset is relatively small, we expect that neural networks would not be the most effective choice, because they typically require larger amounts of data to learn the weights and generalize effectively. Given that our dataset consists mainly of binary variables, the distance metrics utilized for the KNN and SVM models might fail to capture meaningful relationships, which could lead to poor performance. In contrast, ensemble models such as RF and XGB tend to perform well thanks to the diversity they introduce through multiple classifiers. Given the binary nature of our classification task, and assuming there is some linear relationship in the data, logistic regression should also be a good fit for the task.

To date, ML has yet to be studied in the context of gender-affirming surgery. The heterogenous nature of the transgender and gender-diverse patient population poses a unique challenge for physicians, who often lack conclusive validated data that can be effectively extrapolated to the individual patient.

This study is among the first to apply multiple ML models to predict complications specifically in gender-affirming mastectomies. Although previous literature has explored risk factors for complications in this population, predictive modeling remains underdeveloped. Our findings contribute by presenting a structured, internally validated approach to risk stratification in a historically underrepresented surgical cohort.

Our study demonstrates evident strengths, with carefully curated databases, pedantic algorithm engineering and training, and extensive model evaluation and comparison. However, it bears limitations that are worth mentioning, mainly its retrospective evaluation of outcomes, limited sample size, and single-surgeon experience analysis limiting generalizability. Although our sample is relatively large for this niche population, ML models generally benefit from even larger datasets for improved performance, especially neural networks. Moreover, external validation on multi-institutional datasets is necessary to confirm these findings' robustness and applicability.

The prospects of our trained models are real-time evaluation of expected complication risk at the time of patient counseling. Accurate estimation of the risk of complication development can guide preoperative planning, patient counseling, and expectations; all to ensure safety and satisfaction with the outcome.

Integration of ML models such as RF into preoperative evaluation may support surgeons in risk assessment, patient counseling, and surgical planning. For the plastic surgery community, particularly those involved in gender-affirming care, this represents an opportunity to incorporate data-driven decision tools to improve patient safety and optimize outcomes. Future prospective validation could further establish these models as part of routine surgical risk stratification.

## CONCLUSIONS

In this study, the RF algorithm demonstrated the highest predictive performance among the evaluated ML models for anticipating complications after gender-affirming mastectomies. Incorporating such models into preoperative assessment could improve patient counseling, guide surgical planning, and enhance surgical safety and satisfaction. Because of the fact that these findings are based on a single-surgeon, retrospective dataset, validation of these findings on multi-institutional databases is necessary before clinical adoption.

## Supplemental Material

This article contains supplemental material located online at https://doi.org/10.1093/asjof/ojaf143.

## Supplementary Material

ojaf143_Supplementary_Data
