# Supplementary material for: Implementing Machine Learning Models for Prediction of Gender-Affirming Mastectomy Complications: Estimating Performance and Accuracy
Source: Aesthet Surg J Open Forum. 2025 Nov 1;7:ojaf143. doi: 10.1093/asjof/ojaf143 (PMC12683286; doi:10.1093/asjof/ojaf143)
Supplement: ojaf143_Supplementary_Data [file ojaf143_supplementary_data.docx]

**Appendix: Tuned Hyperparameters Used for Final Model Performance Evaluation**

- Logistic Regression: 'C' was tuned to 2.213.
- Random Forest: 'n_estimators' was tuned to 71 and 'max_depth' to 19.
- XGBoost: Adjusted 'n_estimators' was tuned to 81, 'max_depth' to 11, and 'learning_rate' to 0.0225.
- K-Nearest neighbors:  'n_neighbors', was set to 19.
- Neural Networks: The model included three layers with 'n_units_l0' as 183. 'n_units_l1' as 353 and 'n_units_l2' as 201.
- Support Vector Machine: 'C' was tuned to 1.649.
